# Editorial: AI-Enabled Data Science for COVID-19

**DOI:** 10.3389/fdata.2021.802452

**Published:** 2021-11-24

**Authors:** Da Yan, Hong Qin, Hsiang-Yun Wu, Jake Y. Chen

**Affiliations:** ^1^ Department of Computer Science, University of Alabama at Birmingham, Birmingham, AL, United States; ^2^ Department of Computer Science and Engineering, University of Tennessee at Chattanooga, Chattanooga, TN, United States; ^3^ Research Unit of Computer Graphics, Institute of Visual Computing and Human-Centered Technology, TU Wien, Austria; ^4^ Informatics Institute, School of Medicine, University of Alabama at Birmingham, Birmingham, AL, United States

**Keywords:** COVID-19, artificial intelligence, AI, pandemic, data mining

COVID-19 is a pandemic that has swept all over the world. As of this writing, the New York Times reported that the United States has over 45.4 million cases and 736,000 deaths, and the worldwide numbers are over 240 million cases and 4.9 million deaths. New variants of SARS-CoV-2 continue to emerge and can be more infectious, as we witnessed new surges of the Delta variant worldwide in 2021. Therefore, fighting against COVID-19 is a public health topic of paramount importance.

Many COVID-19 related datasets have already been collected, and the rapid advancement of AI and Data Science has created new software tools for researchers to characterize epidemiological and biological characteristics of COVID-19. In this Research Topic that started in mid-2020, we have openly solicited and collected eight articles in this research direction. This Research Topic represents recent advances in computational approaches to epidemiological modeling, risk analysis, precision diagnosis, and disease progression of COVID-19.

Two papers studied the spread of COVID-19, and such epidemiological models are useful to help the authority decide the proper preventive measures such as stay-at-home orders, travel restrictions, school closure, mask-wearing mandate, and so forth. Li et al. proposed a time-dependent SEIR model that considers the incubation period to mathematically describe the dynamic of the COVID-19 pandemic. The model takes immunity, reinfection, and vaccination into account and can monitor the trajectories of changing parameters, such as transmission rate, recovery rate, and the basic reproduction number. Potgieter et al. emphasized the use of mobility data in modeling the COVID-19 spread through the population. Different mobility data sources were compared to provide insight on which data provides what type of information and in what situations a particular data source is the most useful.

Some COVID-19 patients may develop severe pneumonia in both lungs. COVID-19 pneumonia is a serious illness that can be deadly, so a lot of works have merged that conduct computer-aided detection of such patients from chest CT or X-ray images, using the deep learning technology for computer vision. Nguyen et al. raised the concern about the generalizability of such models, given the heterogeneous factors in training datasets. Their study examined the severity of this problem by evaluating deep learning classification models trained to identify COVID-19 positive patients on 3D CT datasets from different countries. The study confirmed that such models cannot easily generalize to an entirely new dataset distribution never seen before due to factors including patient demographics and differences in image acquisition or reconstruction; and the best-performing model for a particular dataset tends to be a model trained on multiple datasets.

Four works studied how to train robust models and/or interpretable models with the electronic health records (EHRs) of COVID-19 patients to predict symptoms, mortality, and other risk factors. Such prediction models would help the planning of medical resources to individuals most at-risk when healthcare services are under high pressure and would help improve the healthcare outcomes of COVID-19 patients in time. To build a cohort-independent robust mortality prediction model, Bai et al. conducted an international, bi-institutional study from China and Germany. A random forest model was applied to 1,352 patients from the Wuhan cohort, which identified five effective clinical features at admission, including lymphocyte, neutrophil count, C-reactive protein, lactate dehydrogenase, and α-hydroxybutyrate dehydrogenase. These features were also found to be robust over time when patients are in the hospital, and the model was found to generalize well on the independent Würzburg cohort. Mamidi et al. developed an interpretable COVID-19 risk calculator for individuals by utilizing de-identified electronic health records (EHR) from UAB-i2b2 COVID-19 repository under the U-BRITE framework. The generated risk scores are analogous to commonly used credit scores where higher scores indicate higher risks for COVID-19 infection. The authors found that within the 2 weeks before a COVID-19 diagnosis, the most predictive features were respiratory symptoms and other chronic conditions; when extending the timeframe to include all medical conditions across all time, their models also uncovered several chronic conditions impacting the respiratory, cardiovascular, central nervous and urinary organ systems. Snider et al. used SHAP (SHapley Additive exPlanations) to study the impacts of various attributes of the COVID-19 patients in an XGBoost model, which was applied to a dataset containing 57,390 individual COVID-19 cases and 2,822 patient deaths in Ontario, Canada. The most important attributes were found to be age, date of the positive test, sex, income, dementia and some others. Jamshidi et al. conducted a comprehensive evaluation of existing machine learning methods, and created two models based solely on the age, gender, and medical histories of 23,749 hospital-confirmed COVID-19 patients from February to September 2020: a symptom prediction model (SPM) and a mortality prediction model (MPM).

Finally, this Research Topic also included a survey paper by Abdulkareem and Petersen, who carefully summarized recent technological tools, artificial intelligence (AI) tools in particular, that have been used in the detection, diagnosis and epidemiological predictions, forecasting and social control for combating COVID-19. The work highlighted areas of successful applications and underscored issues that need to be addressed to achieve significant progress in battling COVID-19 and future pandemics.

